# Towards a Novel Diagnostic Tool for Autoimmune Rheumatic Diseases: Classification of SLE Using FTIR Spectroscopy and Machine Learning

**DOI:** 10.3390/biomedicines14102191

**Published:** 2026-09-28

**Authors:** Emma L. Callery, Sarah Dyball, Camilo L. M. Morais, Jemma V. Taylor, Anastasia-Vasiliki Madenidou, Ihtesham Ur Rehman, Ian N. Bruce, Anthony W. Rowbottom

**Affiliations:** 1 Department of Immunology, Royal Preston Hospital, Preston PR2 9HT, UK; jemma.taylor@lthtr.nhs.uk (J.V.T.);; 2 Division of Musculoskeletal and Dermatological Sciences, Faculty of Biology, Medicine and Health, University of Manchester, Manchester M13 9NT, UK; sarah.dyball@manchester.ac.uk (S.D.); anastasia.madenidou@manchester.ac.uk (A.-V.M.); ian.bruce@manchester.ac.uk (I.N.B.); 3 National Institute for Health Research Manchester Biomedical Research Centre, Manchester University NHS Foundation Trust, The University of Manchester, Manchester M13 9WU, UK; 4 Center for Education, Science and Technology of the Inhamuns Region, State University of Ceará, Tauá 63660-000, Brazil; camilomorais1@gmail.com; 5 School of Medicine and Dentistry, University of Lancashire, Preston PR1 2HE, UK; iurehman@uclan.ac.uk

**Keywords:** systemic lupus erythematosus, FTIR spectroscopy, machine learning, connective tissue disease, vibrational spectroscopy, biomarker, diagnostics

## Abstract

**Background/Objective**: Systemic lupus erythematosus (SLE) is a complex autoimmune disease with clinical and serological heterogeneity. Current laboratory assays show variable performance for disease detection and risk stratification, resulting in diagnostic delays exceeding six years. There is an unmet need for improved diagnostic and monitoring tools. This study examines the clinical application of Fourier-transform infrared (FTIR) spectroscopy as a rapid, label-free technique for classifying SLE and other connective tissue diseases (CTDs). **Methods**: Serum samples from patients with SLE, Sjögren’s syndrome (SS), undifferentiated CTD (UCTD), and healthy controls (HCs) were analysed by FTIR spectroscopy followed by genetic algorithm–linear discriminant analysis (GA-LDA) modelling at two time points: baseline and six-month follow-up. **Results**: FTIR spectroscopy reliably differentiated CTDs from HCs with accuracies of 94.2% (inclusive of all time points), 99.3% at baseline, and 97.5% at follow-up. SLE patients were successfully separated from disease controls, achieving classification accuracies of 100% at baseline and 97.4% at follow-up. Subgroup analysis also demonstrated good classification accuracies for SLE (81.7%), SS (82.9%), and UCTD (86.1%), with discriminatory spectral features aligning with disease-related biochemical assignments. Notably, wavenumbers associated with proteins, carbohydrates, lipids, and DNA showed increased absorbance intensities in CTD patients, potentially reflecting immune dysregulation and metabolic changes. **Conclusions**: FTIR spectroscopy can reliably distinguish patients with SLE from both other CTDs and healthy individuals and may offer greater diagnostic utility than conventional serological testing. Identification of longitudinal variations in wavenumber biomarkers further supports the clinical potential of this technique and suggests possible future applications in therapeutic monitoring and risk stratification within rheumatological autoimmune disorders.

## 1. Introduction

Systemic lupus erythematosus (SLE) is a complex, heterogeneous autoimmune disease for which no diagnostic criteria exist. Whilst classification criteria have been developed for research purposes, they prioritise specificity, so using them in clinical practice can lead to missed or delayed diagnoses, particularly in early, mild, or atypical cases. As a result, patients may see multiple providers and spend months or years seeking an accurate diagnosis. Diagnostic delays and errors have vast consequences for patients with lupus, both mentally and physically.

Conventional laboratory approaches, many of which were established several decades ago, demonstrate variable performance for disease detection and risk stratification. These methods have not kept pace with advances in our understanding of complex immunological disease mechanisms, highlighting a clear need for the development and integration of novel diagnostic technologies.

Analytical approaches capable of capturing systemic biochemical alterations are increasingly being explored in complex diseases such as SLE. Among these, vibrational spectroscopic techniques provide a means of examining the molecular composition of biological samples in a non-targeted manner [1,2]. Rather than quantifying individual biomarkers, these methods detect collective biochemical changes that occur as a result of disease-driven physiological processes.

Fourier-transform infrared (FTIR) spectroscopy is one method of vibrational spectroscopy that measures how infrared radiation interacts with a sample to produce a spectrum reflective of its molecular structure [3]. Variations within this spectrum arise from differences in chemical bond vibrations across a wide range of biomolecules, including proteins, nucleic acids, lipids, and carbohydrates. Changes in these spectral patterns can therefore reflect underlying pathological processes, supporting the use of FTIR as a novel diagnostic approach.

Alongside FTIR, a range of alternative label-free analytical platforms have been explored for biomedical diagnostics, most notably Raman spectroscopy and electrochemical sensing approaches. Raman-based techniques, including surface-enhanced Raman spectroscopy (SERS), offer high molecular specificity and, when combined with nanostructured substrates, can achieve ultra-sensitive detection of biomolecules at extremely low concentrations. Recent reviews by Lin et al. and Zhang et al. 2024 [4,5] have demonstrated the capability of SERS to detect and differentiate analytes within biological specimens using engineered substrates and machine learning approaches. In parallel, electrochemical sensing platforms provide rapid, quantitative, and highly sensitive detection through analyte-specific signal transduction and have been widely applied in point-of-care diagnostics, including biomarker detection in complex biofluids [6]. Nevertheless, these approaches are typically dependent on predefined targets, which may limit their ability to capture broader biochemical changes associated with complex disease states. The widespread adoption of these platforms into routine diagnostic pathways has been further hindered by reproducibility issues and high costs.

In contrast, FTIR spectroscopy enables an untargeted approach, capturing biochemical information across multiple molecular groups within a single measurement [1,2,3]. This feature is a key strength in heterogeneous diseases such as systemic lupus erythematosus, where pathological processes involve widespread immune system dysfunction and multi-organ damage. Moreover, recent advances in chemometric analysis and machine learning have significantly accelerated the clinical potential of vibrational spectroscopy, facilitating the interpretation of complex spectral datasets into reliable diagnostic tools [7]. Fourier-transform infrared (FTIR) spectroscopy has demonstrated utility across a range of clinical applications, as reviewed by Callery and Rowbottom and Kannan et al. [8,9], supporting its diagnostic performance.

Current diagnostic testing for SLE has limitations. Routinely used approaches lack the ability to detect the full repertoire of SLE-associated autoantibodies, further hindered by the fact that these antibodies chemically and structurally change over time. Furthermore, there are well-described methodological variances between current platforms described in the literature, the clinical relevance of which remains unknown; thus, clinicians are faced with a diagnostic dilemma when managing their patients. Delayed or inaccurate diagnosis has significant consequences, leading to postponed treatment and poorer clinical outcomes.

The overarching aim of this work is to advance the clinical translation of vibrational biospectroscopy towards routine use within diagnostic immunology laboratories and, ultimately, near-patient testing in clinical settings. The development of a superior blood test that can reliably differentiate SLE from the healthy population and other connective tissue diseases (CTDs) could provide substantial clinical benefit in both primary and secondary care settings. Herein, we have applied FTIR spectroscopy to build upon our previous work demonstrating the diagnostic potential of vibrational spectroscopy in SLE. We previously applied the complementary method of Raman spectroscopy to classify SLE patients from controls with 99% accuracy, 100% sensitivity and 99% specificity [10]. While Raman-based approaches evaluated in our earlier work showed promising diagnostic performance, their broader clinical translation remains limited by higher instrumentation costs, susceptibility to fluorescence interference and challenges in achieving inter-laboratory consistency. In contrast, FTIR enables low-cost, standardised biochemical profiling, making it more suitable for translation into routine diagnostic settings (Baker et al., 2016 [1]).

In this study, we evaluate the diagnostic role of FTIR spectroscopy using a larger cohort of SLE patient samples and disease controls. We assess spectral differences across two time points to examine potential disease changes over time and include patients with Sjögren’s syndrome (SS) and Undifferentiated Connective Tissue Disease (UCTD) as disease controls. Further, we identified significant disease-specific spectral features which may be developed into future diagnostic biomarkers. As an underutilised platform in immunological disorders, FTIR spectroscopy has far-reaching potential as a diagnostic method, overcoming current diagnostic challenges and improving patient care.

## 2. Materials and Methods

### 2.1. Sample Population

Between 4 May 2022 and 9 November 2022, adult patients were recruited into the Disease Outcomes and Biomarkers across SARDs cohort from Manchester University NHS Foundation Trust, a tertiary SARDs referral centre. Patients were recruited consecutively from new and follow-up connective tissue disease clinics, as previously described [11]. Patients with an established SARD diagnosis of UCTD, SLE and SS were eligible for inclusion if they had ≥1 clinical feature of an SARD and were positive for the anti-Ro (SSA) antibody, measured using the BioPlex 2200 ANA Screen [12]. A clinical diagnosis by a consultant rheumatologist was used to identify eligible patients, and patients were required to meet the classification criteria for their respective disease [13,14,15]. Ethical approval was obtained [REC ID: 21-WM-0235]; all patients signed written informed consent, and the study was conducted in accordance with the Declaration of Helsinki. Healthy controls (HCs) were included from the Lupus Extended Autoimmune Phenotype (LEAP) study [16]. Ethical approval was obtained [REC ID: 13/NW/0564]; all patients signed written informed consent, and the study was conducted in accordance with the Declaration of Helsinki.

### 2.2. Data Collection

Comprehensive clinical data were obtained at the time of participant recruitment and at the 6-month follow-up. Autoantibodies were measured using the BioPlex 2200 ANA Screen with Medical Decision Support Software (MDSS) (comprising the following autoantigens: dsDNA, Chromatin/nucleosome, Ro/SS-A, La/SS-B, Smith (Sm), SmRNP, RNP, Scl-70, Jo-1 and Centromere) [12]. Demographics for each participant were recorded in laboratory information management systems and extracted prior to statistical analysis.

### 2.3. Statistical Analysis

Patient and serological data were exported from laboratory information management systems to Microsoft Excel for analysis. Data comparisons were made using MATLAB R2014b for sex, age, ethnicity, disease activity score (DAS), treatments (anti-malarials and biologic immunosuppressants), and the following antibodies: dsDNA, anti-Ro52, anti-Ro60, anti-La, anti-Sm, anti-RNP, anti-Chromatin, anti-Centromere, anti-Scl70, and anti-Jo-1. Statistical analysis was carried out using a Kruskal–Wallis test, where *p*-values below 0.05 were considered statistically significant.

### 2.4. Acquisition of Spectra by FTIR

Spectral analysis was performed based on published protocols [17]. All samples were stored at −80 °C until processing and underwent a single freeze–thaw cycle prior to FTIR analysis. Frozen serum samples were defrosted and homogenised by vortexing. Spots of 5 µL of serum were placed on a calcium fluoride slide and spread to a diameter of approximately 1 cm. Two slides were prepared per serum sample. The slides were left to air dry for 2 h at 19 °C. All sample preparation and drying procedures were conducted in a temperature-controlled, air-conditioned laboratory environment to minimise the potential influence of environmental variability on spectral acquisition. Samples were tested blindly, in four batches during the period 16 July 2024 and 27 September 2024. Spectra were acquired on the Nicolet Summit Pro FTIR analyser (ThermoFisher, Waltham, MA, USA) at a resolution of 4 cm^−1^ and a wavenumber range of 800–4000 cm^−1^, with 32 scans per acquisition. A blank background reading was measured by analysing an area of the slide with no sample present, and this was subtracted from the measurement of the sample. Each sample was acquired with 6 technical replicates (3 replicates each on 2 slides). To ensure repeatability and minimise experimental variability, each sample was analysed using multiple technical replicates, and spectra were pre-processed using optimised methods established in prior work [18], including smoothing, derivative transformation, and normalisation. These steps, together with control of environmental and experimental factors, have been shown to improve spectral consistency and reproducibility in FTIR analysis of biofluids.

### 2.5. Data Analysis

The FTIR spectra at the biofingerprint region (900–1800 cm^−1^) were analysed within the MATLAB R2014b (MathWorks, Inc., Natick, MA, USA) environment using lab-made routines, the Irootlab toolbox [19], and the Classification Toolbox for MATLAB [20]. The six replica spectra per sample were averaged so the classification analyses were performed on a sample basis. To ensure spectral quality, raw spectra were first visually inspected for artefacts, and signal-to-noise ratios (S/N) were evaluated across representative samples prior to pre-processing. A predefined absorbance threshold was not applied. Instead, spectral quality was assessed through visual inspection for artefacts, signal-to-noise ratio evaluation and objective outlier detection using Hotelling’s T^2^ versus Q residual analysis. The acquired spectra exhibited high absorbance intensity typical of dried serum films, with clear amide I and II features. The S/N values calculated based on the amide I peak (1650 cm^−1^) in relation to the noise (800 cm^−1^) resulted in 10.94 ± 2.75 absorbance units, indicating a 10-fold signal intensity compared to the noise. Thereafter, two outlier spectra were identified and removed from the dataset based on the Hotelling’s T^2^ vs. Q residuals test [7] (Supplementary Information, Figure S1). These outliers show anomalous spectra with great suppression of amide I and II peaks, which may be caused by errors during sample preparation or when handling the samples during spectral acquisition. The data were then pre-processed by Savitzky–Golay smoothing (window of 15 points, 2nd order polynomial fitting) to reduce random noise, 2nd derivative to correct the baseline and enhance small spectral differences, and vector normalisation to reduce possible experimental variations such as sample thickness [7]. Six discriminatory comparisons between the samples were performed in the dataset: (1) HC (n = 10) vs. all CTDs (inclusive of all time points) (n = 56); (2) HC (n = 10) vs. all CTD baselines (n = 28) vs. all CTD follow-ups (n = 28); (3) primary subgroup analysis for SLE/SLE overlap syndrome (n = 14) vs. other CTDs (n = 14) for baseline samples; (4) primary subgroup analysis for SLE/SLE overlap syndrome (n = 16) vs. other CTDs (n = 12) for follow-up samples; (5) secondary subgroup analysis for SLE/SLE overlap (n = 14) vs. SS (n = 8) vs. UCTD (n = 6) for baseline samples; and (6) secondary subgroup analysis for SLE (n = 16) vs. SS (n = 7) vs. UCTD (n = 5) for follow-up samples.

The pre-processed spectral data underwent chemometric analysis by genetic algorithm with linear discriminant analysis (GA-LDA) [21], which was shown to be the best discriminatory algorithm among other algorithms tested in comparison (1) (Supplementary Information, Table S1). Due to the small number of samples, the GA-LDA models were built using all the available samples and validated by Monte Carlo cross-validation using 1000 iterations with 20% of samples left out for validation [10]. The genetic algorithm (GA) is a feature selection algorithm inspired by Mendelian genetics [22], where a set of initial variables (e.g., wavenumbers) undergo processes such as selection, cross-over and mutation until the best set of variables according to a fitness function is found [7]. The GA fitness function is calculated by minimising the cost function G:

(1) $$G=\frac{1}{N_{V}}\sum_{n=1}^{N_{V}}g_{n}$$

where $N_{V}$ represents the number of validation samples and $g_{n}$ is calculated by:

(2) $$g_{n}=\frac{r^{2}\left(x_{n},M_{I(n)}\right)}{{min}_{I(m)\neq I(n)}r^{2}\left(x_{n},M_{I(m)}\right)}$$

where $r^{2}\left(x_{n},M_{I(n)}\right)$ is the squared Mahalanobis distance between the sample $x_{n}$ and the centre of its true class $M_{I(n)}$, and $r^{2}\left(x_{n},M_{I(m)}\right)$ is the squared Mahalanobis distance between the sample $x_{n}$ and the centre of the closest wrong class $M_{I(m)}$ [21]. In this study, GA was performed using 100 generations with 200 chromosomes each. Mutation and cross-over probabilities were set at 10% and 60%, respectively [23].

The linear discriminant analysis (LDA) is then applied to the selected variables by GA, for which the classification scores are calculated as follows:

(3) $$L_{ik}={\left(x_{i}-{\bar{x}}_{k}\right)}^{T}C_{pooled}^{-1}\left(x_{i}-{\bar{x}}_{k}\right)$$

where $L_{ik}$ is the LDA classification score, $x_{i}$ is a vector containing the input variables for sample i, ${\bar{x}}_{k}$ is the mean vector of class k and $C_{pooled}$ is the pooled covariance matrix between the investigated classes [21].

Model performance was assessed using Monte Carlo cross-validation, an exhaustive iterative algorithm where a random portion of the data (20%, in this case) is set apart in a temporary validation set and predicted using the remaining training data (80%) [24]. Model validation is ensured by calculating the mean accuracy (AC), sensitivity (SENS) and specificity (SPEC) during this process [7]. These quality metrics are calculated as follows:

(4) $$AC\left(\%\right)=\left(\frac{TP+TN}{TP+FP+TN+FN}\right)\times 100$$

(5) $$SENS\left(\%\right)=\left(\frac{TP}{TP+FN}\right)\times 100$$

(6) $$SPEC\left(\%\right)=\left(\frac{TN}{TN+FP}\right)\times 100$$

where TP stands for true positives, TN for true negatives, FP for false positives and FN for false negatives.

Wavenumbers responsible for class separation were identified on the 2nd derivative spectrum to enhance small spectral differences. The discriminatory wavenumbers identified by GA-LDA are model-specific and correspond to variables contributing most strongly to class separation. Therefore, different disease-group comparisons may generate distinct spectral biomarkers despite similarities in overall spectral appearance, reflecting the unique biochemical differences captured within each model. Furthermore, receiver operating characteristic (ROC) curves and their area under the curve (AUC) values were calculated for both training and Monte Carlo cross-validation. The AUC is a metric to evaluate the model’s ability to discriminate the samples, where AUC ≤ 0.5 suggest no discrimination, between 0.7 and 0.8 suggests acceptable discrimination, between 0.8 and 0.9 suggests excellent discrimination, and above 0.9 suggests outstanding discrimination [10].

## 3. Results

The clinical characteristics of the patients (n = 30) recruited were collected by physicians using medical records where required, summarised in Table 1. The diagnosis of CTD and clinical classification of patients into subgroups were made according to 1997 ACR criteria for SLE and the 2016 ACR/EULAR criteria for SS at the discretion of a trained rheumatologist. Patients diagnosed as UCTD did not meet classification criteria at recruitment for a definitive CTD, but had clinical and laboratory findings typical for CTD. Of the 30 patients recruited, two SLE patients in the baseline cohort were identified as outliers (as described in Section 2.5). In the follow-up cohort, samples were not provided for two patients (1 SS and 1 UCTD).

Patient demographic data were compared as outlined in the Methods section to identify variables that might influence subgroup discrimination. Statistical analysis identified significant differences between disease groups for immunosuppressant use and anti-Sm antibody positivity (*p* < 0.05; Supplementary Information, Table S2). However, the present study was not sufficiently powered to determine the independent contribution of these variables to spectral discrimination. Additional demographic differences were observed between patient groups (Table 1). Specifically, individuals of Black ethnicity were overrepresented within the UCTD cohort, anti-La antibodies were more prevalent in patients with SS, and anti-Sm antibodies were more frequently observed in patients with UCTD. No other statistical differences were observed based on the demographic data, although some borderline associations may be present when evaluating anti-RNP and anti-Chromatin manifestations (more frequent in SLE/SLE overlaps) and anti-malarial medication, which is more frequent in SLE/SLE overlap follow-ups.

### 3.1. HCs (n = 10) vs. All CTD Patients (n = 56); Baseline (n = 28) vs. Follow Ups (n = 28)

We performed an analysis of HC vs. all CTD patients, inclusive of all time points. The six replica spectra per sample were pre-processed and averaged (Figure 1a) so that all classification analyses were performed on a sample basis. The spectra were analysed in the biofingerprint region (900–1800 cm^−1^), which is a key region of the IR spectrum that contains several vibrations of functional groups in important biomolecules such as proteins, lipids, carbohydrates and amino acids [7,25]. Chemometric analysis of pre-processed spectra by GA-LDA and Monte Carlo cross-validation showed excellent discrimination between HC and all CTD patients (Figure 1b). For HC classification, diagnostic performance was assessed and showed an accuracy of 94.2%, sensitivity of 90.2% and specificity of 98.4%. For CTD classification, the model demonstrated an accuracy of 94.2%, sensitivity of 98.4% and specificity of 90.2%. In the analysis of HC vs. all CTD (baseline) vs. all CTD (follow-up) patient groups, replica spectra were pre-processed and averaged (Figure 1c) prior to chemometric analysis. GA-LDA and Monte Carlo cross-validation showed excellent discrimination between HC, all CTD patients at baseline analysis, and all CTD patients at follow-up (Figure 1d). Quality metrics were calculated for the GA-LDA model, as shown in the confusion matrix for the Monte Carlo validation data (Figure 1e). For HC classification, accuracy, sensitivity, and specificity of the validation set were 95.2%, 92.1% and 98.4%, respectively; for all CTD baseline patients, 99.3%, 99.1% and 99.6%, respectively; and for all CTD follow-up patients, 97.5%, 97.1% and 97.9%, respectively. The 22 wavenumbers responsible for class separation were identified on the second derivative spectrum to enhance small spectral differences (Figure 1f). Receiver operating characteristic (ROC) curve analysis with AUC values was calculated for HC, all CTD baseline and all CTD follow-up at 0.953, 0.993 and 0.975, respectively (Figure 1g–i).

Band assignments referenced in Table 2, Table 3 and Table 4 are tentative and based on the established literature [26]. Due to the complex composition of serum, individual wavenumbers reflect overlapping contributions from multiple biomolecular classes rather than specific molecular species. The absorbance for all selected wavenumbers by the GA-LDA model was higher for the CTD follow-up group and lower for the HC group (Supplementary Information, Table S3). Using the GA-LDA model, we found 11 protein-associated wavenumbers (1268, 1323, 1348, 1570, 1608, 1615, 1631, 1653, 1674, 1679, and 1685 cm^−1^), corresponding primarily to the amide I and amide II regions. These bands reflect C=O stretching and N-H bending/C-N stretching in the protein backbone and are indicative of overall protein content and structure. We identified five carbohydrate-associated biomarkers (975, 1016, 1061, 1140, and 1154 cm^−1^), representing C-O, C-C, and C-O-C vibrations typical of carbohydrates. Lipid-associated wavenumbers (1438, 1442, 1454, and 1712 cm^−1^) were associated with CH_2_ bending and ester C=O stretching, indicative of fatty acids and esterified lipids. Additionally, wavenumbers at 1189 and 1196 cm^−1^ were associated with phosphate-containing biomolecules (nucleic acids and phospholipids), primarily reflecting asymmetric PO_2_^−^ stretching vibrations.

### 3.2. Primary Patient Subgroup Analysis. Baseline: SLE and SLE Overlap Syndrome (SLEs) (n = 14) vs. Other CTDs (n = 14); Follow-Up: SLE and SLE Overlap Syndrome (SLEs) (n = 16) vs. Other CTDs (n = 12)

In the primary subgroup analysis of CTD patients, classification was performed on a sample basis for patients with SLE and SLE overlap syndrome (SLEs) vs. other CTDS (SS, UCTD). Averaged spectra (second derivative + vector normalisation) per class were compared for baseline and follow-up time points (Figure 2a and Figure 2b, respectively). GA-LDA classification modelling with Monte Carlo cross-validation showed excellent discrimination between SLE and other CTDs at baseline and follow-up analysis (Figure 2c and Figure 2d, respectively). Quality metrics were calculated for the training and validation sets. At baseline for SLE patients, Monte Carlo validation classification accuracy, sensitivity and specificity were 100%, 100% and 100%, respectively, and for other CTD baseline patients, 100%, 100% and 100%, respectively. At the follow-up time point for SLE patients, validation classification accuracy, sensitivity and specificity were 97.4%, 96.1% and 98.7%, respectively, and for other CTD baseline patients, 97.9%, 98.7% and 97.1%, respectively.

The 12 wavenumbers responsible for class separation at each time point were identified on the second derivative spectrum to enhance small spectral differences and plotted on the original spectrum for baseline and follow-up time points (Figure 2e and Figure 2f, respectively). ROC curve analysis with AUC values was calculated for baseline and follow-up at 1.00 and 0.974, respectively. Tentative molecular assignments for biomarkers responsible for separation of SLE and other CTDS patients are referenced in Table 3 for baseline and follow-up analysis [26]. At both time points, the absorbance for all selected wavenumbers by the GA-LDA model was higher for the other CTDs group compared to SLE patients (Supplementary Information, Tables S4 and S5).

At baseline, twelve discriminatory wavenumbers were identified, spanning nucleic acids, carbohydrates, proteins, and lipids. Three DNA-associated peaks (903, 907, and 912 cm^−1^) correspond to phosphodiester backbone vibrations. Carbohydrate-associated bands (1125, 1144, and 1339 cm^−1^) reflect C-O, C-O-C, and ring vibrations characteristic of carbohydrate structures. Protein contributions were represented by the amide I band at 1662 cm^−1^, associated with protein backbone vibrations, predominantly from serum proteins such as albumin, together with an overlapping band at 1233 cm^−1^ indicating contributions from both amide III vibrations and phosphate-containing biomolecules. Lipid-associated features (1349, 1453, 1723, and 1775 cm^−1^) correspond to CH_2_/CH_3_ deformation and ester carbonyl (C=O) stretching vibrations.

In the follow-up data, a further twelve discriminatory wavenumbers were identified, again encompassing all major biomolecular classes. Three DNA-associated wavenumbers (951, 1081, and 1202 cm^−1^) correspond to phosphate-related vibrations, including symmetric and asymmetric PO_2_^−^ stretching. Carbohydrate-associated bands (984, 985, and 990 cm^−1^) represent C-O and C-C vibrations typical of carbohydrate structures. Protein-associated contributions were more prominent at follow-up, with four wavenumbers (1253, 1300, 1353, and 1576 cm^−1^) corresponding to amide III vibrations, protein side-chain modes, and the amide II band. Lipid-associated features (1442 and 1766 cm^−1^) correspond to CH_2_ bending and ester carbonyl stretching vibrations.

### 3.3. Secondary Subgroup Analysis. Baseline: SLE (n = 14) vs. SS (n = 8) vs. UCTD (n = 6) for Baseline Samples; Follow-Up: SLE (n = 16) vs. SS (n = 7) vs. UCTD (n = 5)

In the secondary subgroup analysis of patients with SLE and SLE overlap syndrome (SLEs) vs. SS vs. UCTD, classification was performed on a sample basis. GA-LDA classification modelling with Monte Carlo cross-validation showed discrimination between SLE, SS and UCTD patients at baseline and follow-up time points (Figure 3a and Figure 3b, respectively). Performance was assessed for the Monte Carlo cross-validated GA-LDA model; at baseline, it demonstrated accuracy, sensitivity and specificity at 73.4%, 69.4% and 77.8%, respectively, for SLE; 82.9%, 78.8% and 87.4%, respectively, for SS; and 74.6%, 64.4% and 88.7%, respectively, for UCTD (Figure 3c). At follow-up, we demonstrated accuracy, sensitivity and specificity at 81.7%, 84.3% and 79.4%, respectively, for SLE; 69.4%, 55.2% and 93.4, respectively, for SS; and 86.1%, 83.6% and 88.7%, respectively, for UCTD (Figure 3d).

The wavenumbers responsible for class separation were identified on the second derivative spectrum; six wavenumbers were identified for baseline analysis and five for follow-up (Figure 3e and Figure 3f, respectively). ROC curve analysis with AUC values was calculated for the Monte Carlo cross-validated GA-LDA model; for baseline data, AUC values for SLE were 0.729, for SS 0.601, and for UCTD 0.715 (Figure 3g). At follow-up, AUC values for SLE were 0.818, for SS 0.627, and for UCTD 0.869 (Figure 3h).

Tentative molecular assignments for biomarkers responsible for separation of SLE, SS and UCTD at baseline and follow-up time points are referenced in Table 4 [26]. At baseline, six discriminatory wavenumbers were identified across nucleic acids, carbohydrates, proteins, and lipids. Nucleic acid-associated bands at 933 and 956 cm^−1^ correspond to phosphate-related vibrations, while overlapping contributions from carbohydrates and phosphate-containing biomolecules were observed at 1205 cm^−1^ and from proteins and nucleic acids at 1008 cm^−1^. A mixed lipid/protein signal at 1348 cm^−1^ reflects CH_2_ wagging, and a lipid-associated band at 1774 cm^−1^ corresponds to ester carbonyl (C=O) stretching. At follow-up, five wavenumbers were identified spanning nucleic acid, carbohydrate, and protein classes. Nucleic acid-associated vibrations (927, 948, and 1231 cm^−1^) reflect phosphodiester and phosphate stretching modes, while carbohydrate contributions at 1045 cm^−1^ correspond to C-O stretching. Protein features were represented by the amide I band at 1629 cm^−1^, with no lipid-associated bands detected at follow-up.

At baseline, the absorbance for all six wavenumbers selected by the GA-LDA model was higher for the SS group compared to SLE patients and UCTD (Supplementary Information, Table S6). At follow-up, the absorbance for all selected wavenumbers was higher for the Primary SS group, with the exception of 1629 cm^−1^, which was higher for UCTD (Supplementary Information, Table S7).

## 4. Discussion

Using a blood-based spectroscopic approach, this study demonstrates the capability of FTIR to distinguish SLE patients from both healthy individuals and other CTDs. Furthermore, FTIR spectroscopy detected spectral differences within CTD patients between baseline and 6-month follow-up, enabling subgroup classification at both time points. The high performance of the GA-LDA classification model strengthens our prior findings using Raman spectroscopy for SLE classification [10]. Although Raman spectroscopy achieved excellent classification performance in our previous study, FTIR is better placed for routine clinical use given its lower cost, faster analysis, and greater availability in diagnostic laboratories. Together, our data support the potential of vibrational spectroscopy as a clinically relevant platform for disease screening, classification and monitoring.

Autoantibodies remain central to the diagnosis and classification of SLE, associated with the underlying clinical and immunological heterogeneity of the disease [27]. Anti-dsDNA antibodies, found in 70–98% of patients, are highly specific (up to 97.4%) and thus remain a key biomarker despite concerns about assay variability and variable association with disease pathogenesis [28,29].

However, not all patients with SLE present with anti-dsDNA antibodies. Data from the Lupus Extended Autoimmune Phenotype (LEAP) cohort suggest that less than 50% of patients are dsDNA-positive [16], highlighting the limitations of using this biomarker alone. In clinical practice, two main laboratory methods are used to detect anti-dsDNA antibodies: enzyme-linked immunosorbent assay (ELISA) and the Crithidia luciliae immunofluorescence test. However, differences in results between these methods highlight issues with reliability and, consequently, clinical usefulness. These uncertainties can contribute to significant delays in diagnosis, sometimes extending beyond six years [30]. This highlights a clear unmet need for new approaches that better capture the biochemical heterogeneity of SLE, particularly for patients who do not present with typical serological markers such as anti-dsDNA antibodies.

FTIR spectroscopy is a form of vibrational spectroscopy capable of detecting metabolic changes associated with a variety of immunological disorders, including several cancers, reviewed in detail by Callery and Rowbottom (2021); Kannan et al. (2023) [8,9]. Herein, FTIR spectroscopy combined with chemometric analysis was applied to distinguish SLE patients from both healthy individuals and disease controls. The spectral features driving this separation likely reflect metabolic changes caused by the autoimmune pathogenesis of CTDs. Importantly, these spectral differences appear to be specific to SLE and distinguish it from other CTDs.

The GA-LDA results described outstanding classification performance between the total CTD cohort (SLE, Sjögren’s syndrome (SS), Undifferentiated CTD (UCTD)) and HCs, with clear segregation of CTD patients at baseline and at the 6-month time point. Between the three subgroups, we reported an average classification accuracy of 97%, sensitivity of 96% and specificity of 99%. To interpret FTIR spectral differences in biomedical studies and assign molecular associations which may contribute to disease-specific changes, researchers can use published literature databases and libraries [26]. Importantly, the discriminatory wavenumbers identified by the GA-LDA models differed between disease-group comparisons. This is expected, as the genetic algorithm selects spectral variables that contribute most strongly to class separation within each comparison, reflecting the specific biochemical differences present between the groups rather than identifying a universal set of biomarkers across all CTDs. Furthermore, the overall spectral profiles of biological fluids such as serum are expected to be broadly similar because all individuals share a common biochemical matrix composed predominantly of proteins, lipids, nucleic acids, carbohydrates and other circulating metabolites. Consequently, many spectral features are conserved across samples, and disease-related alterations may be subtle and not readily apparent by visual inspection alone. The GA-LDA approach is specifically designed to identify those spectral regions that contribute most strongly to discrimination, enabling detection of biologically relevant differences despite substantial overlap in the overall spectral appearance. We identified clear spectral differences between CTD patients and HC, with several key discriminatory wavenumbers associated with protein molecules at 1268, 1323, 1348, 1570, 1608, 1615, 1631, 1653, 1674, 1679, and 1685 cm^−1^. These FTIR peaks primarily correspond to the amide I and amide II bands, which reflect C=O stretching and N-H bending/C-N stretching in the protein backbone, key indicators of protein content and secondary structure, including α-helices and β-sheets. Of interest, all wavenumbers demonstrated higher absorbance intensities in the CTD group compared to the HC; we postulate this likely reflects the chronic immune dysregulation associated with these rheumatological disorders, including poorly controlled autoimmune responses, complement activation, interferon dysregulation, and associated inflammation [31]. These features are common across diseases like SLE, mixed connective tissue disease (MCTD), and Sjögren’s syndrome (SS), where patients frequently exhibit hypergammaglobulinemia, increased pro-inflammatory cytokine expression, and evidence of systemic involvement [27,29,32,33,34].

We identified five carbohydrate-associated wavenumbers (975, 1016, 1061, 1140, and 1154 cm^−1^), corresponding to C-O, C-C, and C-O-C vibrations. Carbohydrate-related serological markers are increasingly recognised in autoimmune diseases for their roles in cell signalling, immune recognition and acute-phase responses. Glycosylated proteins and glycosylation patterns are emerging as valuable biomarkers and therapeutic targets in inflammatory disease [35,36]. In SLE, glycosylation of IgG is a known contributor to disease pathogenesis, with recent studies demonstrating subgroup-specific glycosylation patterns [37]. While glycomic profiling shows promise, its high cost and complexity limit clinical uptake. In contrast, FTIR spectroscopy offers a rapid, cost-efficient method to assess such post-translational modifications (PTMs), as supported by our current findings and prior Raman-based work identifying protein phosphorylation signatures in SLE [10].

We also identified four lipid-associated wavenumbers (1438, 1442, 1454, and 1712 cm^−1^), reflecting CH_2_ bending and C=O ester stretching in fatty acids and esterified lipids. Dysregulated lipid metabolism is increasingly implicated in SLE pathogenesis and monitoring. Altered levels of HDL, LDL, triglycerides, and VLDL have been linked to disease activity and cardiovascular risk [38,39]. Additional lipid-related biomarkers such as oxLDL, anti-oxLDL antibodies, and sphingolipids correlate with vascular inflammation, immune signalling, and organ involvement [38,40,41,42]. Currently, the clinical application of lipidomics remains restricted by cost and standardisation issues. FTIR spectroscopy provides a potential alternative for capturing these lipid-related changes.

In this study, we identified two nucleic acid-associated wavenumbers (1189 and 1196 cm^−1^), each with increased absorbance intensity in CTD patients compared to HCs. These findings support our earlier work using Raman spectroscopy, which also showed elevated DNA signals in SLE patients [10]. The clinical utility of measuring nucleic acid-associated biomarkers such as cell-free circulating DNA (cf-DNA) and mitochondrial DNA has previously been investigated, becoming increasingly recognised as mediators of inflammation in autoimmune diseases, including SLE and SS, where they activate innate immune pathways like TLR9 and promote type I interferon responses [43,44,45]. Elevated levels of cfDNA have been shown to correlate with disease activity and tissue inflammation in SLE [46] and SS [47]. To detect these biomarkers, highly sensitive analytical techniques such as quantitative PCR and fluorometric assays are required; however, the cost and complexity of these tests limit their routine use. FTIR spectroscopy is a label-free, low-cost technique that requires minimal sample preparation, making it a practical approach for detecting nucleic acid-associated changes in SLE and related CTDs.

Of further interest, the absorbance intensity of all key spectral wavenumbers was higher in the 6-month follow-up CTD subgroup compared to the baseline. This likely suggests continued inflammatory changes and increased pro-inflammatory processes involving proteins, carbohydrates, lipids, and DNA molecules over time in these patient groups. Of clinical relevance and future impact, in our patient cohort, six patients (two SLE/SLE overlap, three SS, one UCTD) were classified as having a high DAS in the baseline group, which increased to eleven patients (seven SLE/SLE overlap, three SS, one UCTD) in the 6-month follow-up. Whilst the present study was not designed to directly evaluate associations between FTIR-derived spectral biomarkers and clinical disease activity measures, the observed longitudinal changes in wavenumber biomarkers, together with the increase in high disease activity cases at follow-up, suggest that FTIR spectroscopy may be sensitive to disease-related biochemical changes over time. Future longitudinal studies incorporating established disease activity measures, including SLEDAI-2K scores, complement concentrations and autoantibody titres, will be important to determine the potential role of FTIR spectroscopy in disease monitoring and therapeutic response assessment.

Our SLE-focussed primary analysis distinguishing SLE/SLE-overlap from other CTDs (SS and UCTD) generated excellent results using GA-LDA, with 100% accuracy, sensitivity, and specificity at baseline, and 97.4% accuracy, 96.1% sensitivity, and 98.7% specificity at follow-up. At baseline, 12 discriminatory wavenumbers were identified, including carbohydrates (1125, 1144, 1339 cm^−1^), lipids (1349, 1453, 1723, 1775 cm^−1^), and nucleic acids (903, 907, 912 cm^−1^), with only one protein-associated marker (1662 cm^−1^) and an overlapping nucleic acid/protein-associated wavenumber at 1233 cm^−1^. All biomolecular peak intensities were higher in the ‘other CTDs’ group, suggesting overlapping protein features among CTDs but more distinct carbohydrate, lipid, and DNA signatures in SLE. At follow-up, discriminatory wavenumbers spanned all biomolecule classes, including proteins (1253, 1300, 1353 cm^−1^), carbohydrates (984–990 cm^−1^), lipids (1442, 1766 cm^−1^), and nucleic acids (951, 1081, 1202, 1576 cm^−1^). The follow-up group had five additional SLE patients with high DAS compared to the baseline group, in which two patients had high DAS (which continued into the follow-up group). These findings may also suggest an association with changes in disease activity, further supporting the use of FTIR spectroscopy for disease monitoring.

We also performed a secondary analysis to assess whether FTIR spectroscopy could differentiate between distinct CTDs (SLE/SLE overlap, SS, and UCTD). At baseline, GA-LDA showed good subgroup clustering and classification metrics for SLE (73.4% accuracy), SS (82.9%), and UCTD (74.6%). Follow-up analysis demonstrated improved performance for SLE (81.7%) and UCTD (86.1%), though SS showed lower sensitivity (55.2%) despite high specificity (93.4%). The lower AUC values observed likely reflect the challenge of differentiating closely related CTDs, where considerable overlap in clinical manifestations and autoantibody profiles may result in similar underlying biochemical patterns.

We acknowledge that the findings of this study are limited by a small subgroup sample size. However, our findings demonstrate the potential of FTIR spectroscopy to detect biochemical changes beyond the reach of conventional serological assays for CTDs. Further, inclusion of a mixed-ethnicity population demonstrates that our results are applicable across a diverse ethnic population.

Treatment exposure represents an important consideration in spectroscopic analyses of biofluids. In the present cohort, immunosuppressive medication use differed significantly between disease groups, with greater use observed in patients with SLE/SLE overlap syndrome than in SS or UCTD. Whilst classification performance remained high across both baseline and follow-up analyses, the study was not sufficiently powered to perform robust medication-stratified modelling, and therefore the potential influence of treatment on the observed spectral profiles was not assessed independently. Nevertheless, the consistency of the classification results across multiple disease-group comparisons and time points suggests that disease-associated biochemical differences contribute substantially to the observed spectral discrimination. Future studies involving larger cohorts and longitudinal treatment monitoring will be important to further investigate the relationship between therapeutic interventions and FTIR spectral signatures, including their potential value for treatment response assessment.

Current autoantibody-based diagnostics, while critical for SLE classification, have well-documented weaknesses. Moreover, current techniques do not have the capacity to reveal underlying functional or pathophysiological mechanisms. The ANA test, performed via indirect immunofluorescence on HEp-2 cells, offers high sensitivity (95–98%) but low specificity (~57–60%) due to positivity in other autoimmune diseases and healthy individuals [48]. Anti-dsDNA antibodies are highly specific for SLE (95–98%) but show only moderate sensitivity (50–70%) and may fluctuate independently of clinical activity [49,50]. Anti-Sm antibodies are highly specific (~99%) but have low sensitivity (20–30%) and limited value in monitoring disease activity [51]. Similarly, anti-Ro/SSA, anti-RNP, and antiphospholipid antibodies contribute diagnostically or prognostically, but their relative stability limits use in short-term disease monitoring [52,53].

As current approaches to CTD diagnosis and classification do not fully capture the heterogeneity observed within and between diseases, additional tools are needed to better define disease subtypes, support treatment decisions and reduce diagnostic delay. FTIR spectroscopy is a promising, clinically viable option. This technique requires minimal patient sample (~five microlitres) to generate a biochemical profile which may reflect differences in disease phenotype, activity and treatment response. FTIR spectroscopy may also be useful for investigating factors that contribute to disease variability, including sex, ethnicity and underlying pathogenic mechanisms. Combining FTIR-derived spectral data with genomic, transcriptomic and proteomic datasets may provide further insight into the biological processes underlying CTDs and support biomarker identification. While further validation is required, FTIR spectroscopy has the potential to complement existing immunological testing for the diagnosis and monitoring of autoimmune disease.

## 5. Conclusions

SLE remains a challenging disease to diagnose because of its heterogeneous clinical presentation and variable serological findings, which can contribute to substantial diagnostic delays. FTIR spectroscopy offers a rapid, cost-effective and label-free approach for assessing biochemical changes in biofluids, with the ability to detect alterations associated with immune dysregulation and inflammation. In this study, FTIR spectroscopy combined with chemometric analysis successfully distinguished patients with SLE from both healthy controls and individuals with other autoimmune CTDs using serum samples. To our knowledge, this is the first study to demonstrate discrimination of SLE from multiple CTD groups using FTIR spectroscopy. In addition, FTIR spectroscopy detected temporal changes in serum biochemical profiles of patients with CTDs. These findings suggest that FTIR spectroscopy identifies detailed biochemical changes associated with disease status that would not be possible by conventional autoantibody testing alone. While further validation in larger and more diverse cohorts is required, FTIR spectroscopy may complement existing immunological testing for the diagnosis and monitoring of autoimmune disease.

## Figures and Tables

**Figure 1:**
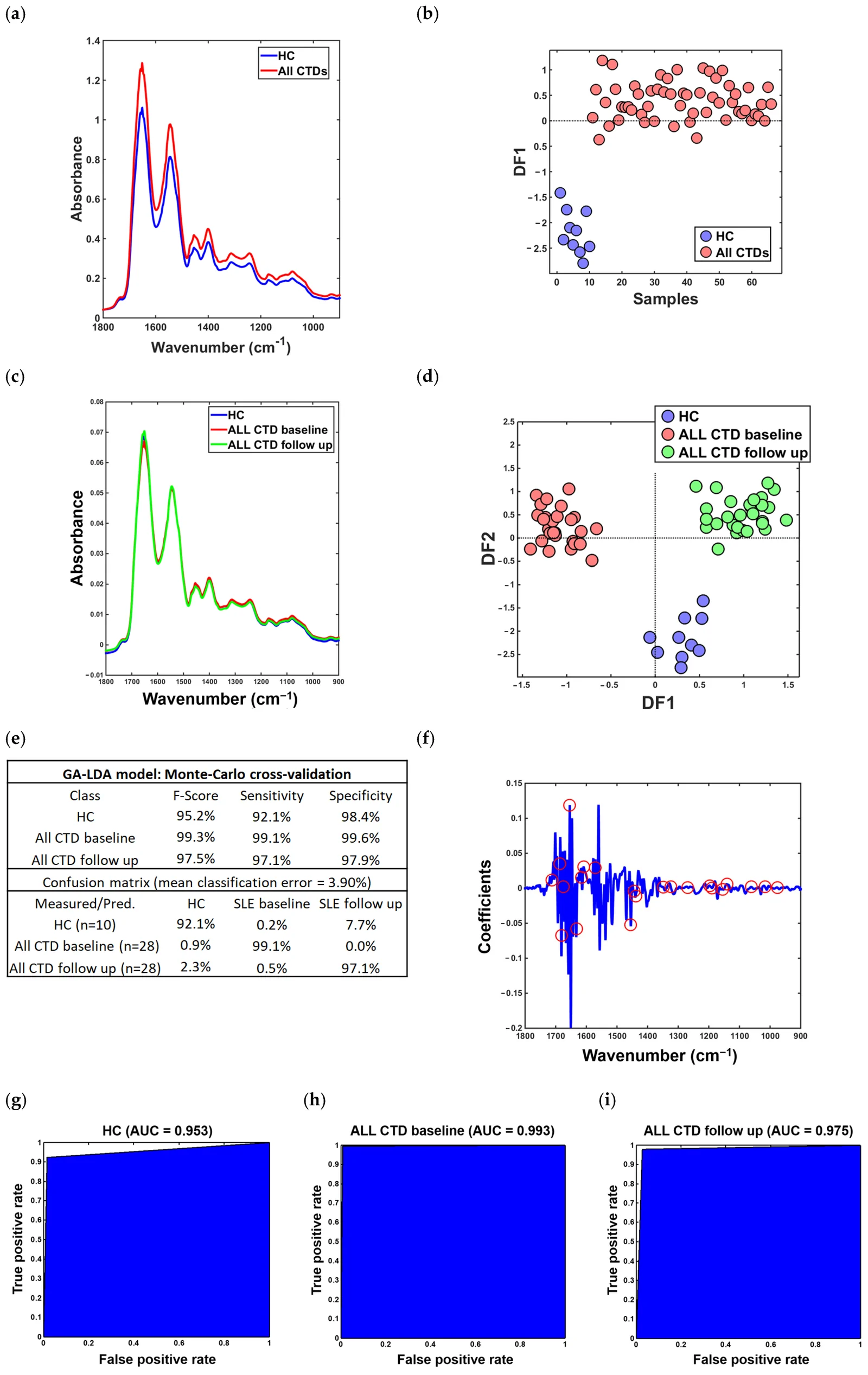
FTIR spectral analysis of healthy controls (HC) vs. all connective tissue disease (CTD) patients (inclusive of all time points), and HC vs. all CTD at baseline vs. all CTD patients at follow-up. (**a**) Averaged fingerprint spectra of HC vs. all CTD patients (inclusive of all time points). (**b**) Genetic algorithm with linear discriminant analysis (GA-LDA) model showing excellent separation between groups; DF is the discriminant function showing the scores on canonical variable 1. (**c**) Averaged fingerprint spectra of HC vs. all CTD at baseline vs. all CTD patients at follow-up. (**d**) GA-LDA model showing excellent separation between groups, DF are the discriminant functions showing the scores on the canonical variables 1 and 2. (**e**) GA-LDA confusion matrix: accuracy (F-score), sensitivity and specificity calculated for the Monte Carlo cross-validation data were 95.2%, 92.1% and 98.4%, respectively, for HC; 99.3%, 99.1% and 99.6%, respectively, for SLE baseline; and 97.5%, 97.1% and 97.9%, respectively, for SLE follow up. (**f**) Key wavenumbers (red circles) identified on 2^nd^ derivative spectrum by GA-LDA. (**g**–**i**) Receiver operating characteristic (ROC) curve analysis using Monte Carlo cross-validation showed areas under the curve (AUC) of 0.953 for HC, 0.993 for CTD baseline, and 0.975 for CTD follow-up patients.

**Figure 2:**
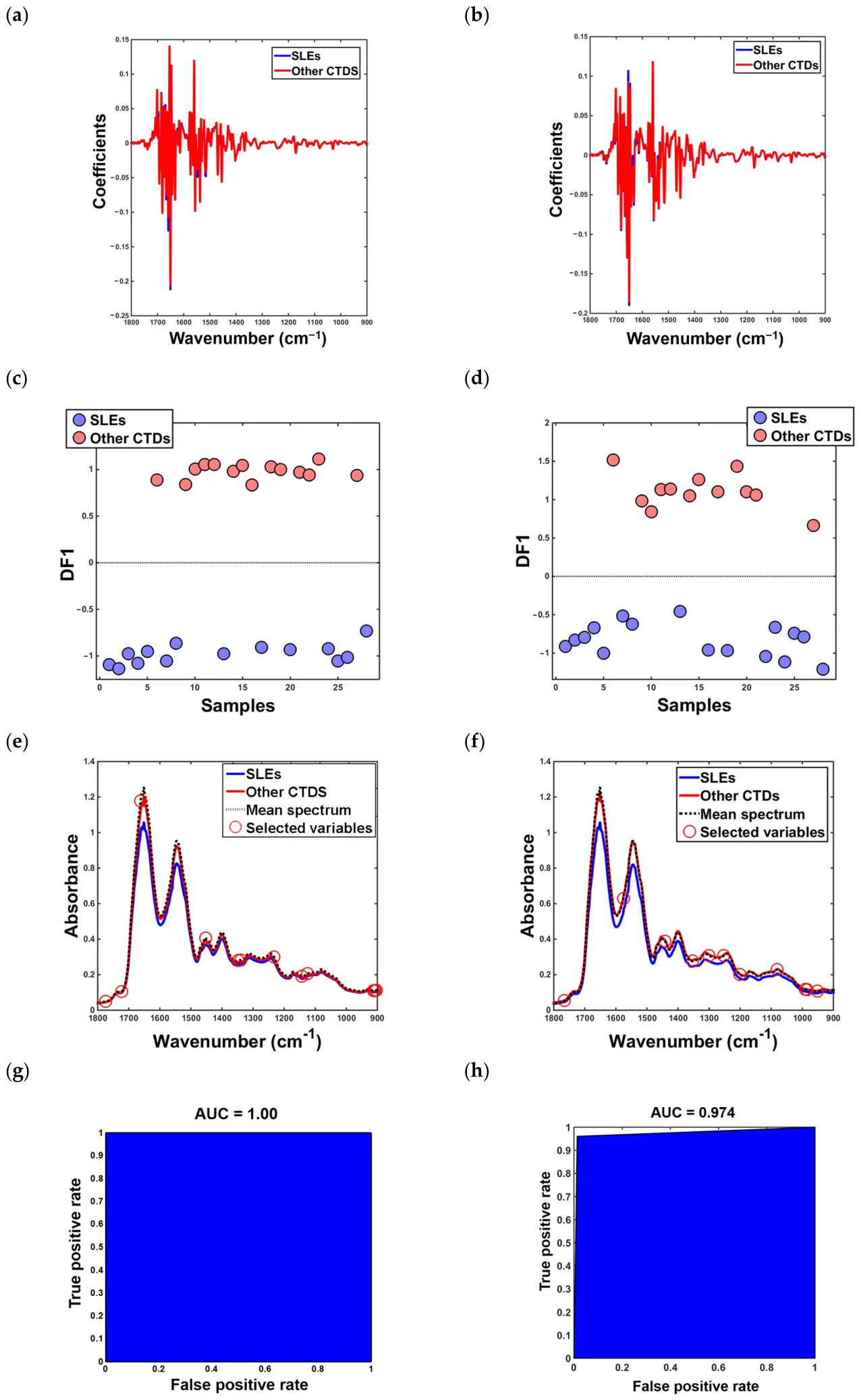
FTIR spectral analysis of systemic lupus erythematosus (SLE) and SLE overlap (SLEs) vs. other CTD patients (primary Sjögren’s syndrome (SS) and undifferentiated CTD (UCTD)) at baseline and at follow-up time points. (**a**) Averaged 2nd derivative, vector-normalised fingerprint spectra at baseline. (**b**) Averaged 2nd derivative, vector-normalised fingerprint spectra at follow-up. (**c**) GA-LDA model (Monte Carlo cross-validated) showing excellent separation between groups at baseline. For SLEs, accuracy, sensitivity and specificity were 100%, 100% and 100%, respectively, and for other CTD baseline patients, 100%, 100% and 100%, respectively. (**d**) GA-LDA model (Monte Carlo cross-validated) showing excellent separation between groups at follow-up. For SLEs, accuracy, sensitivity and specificity were 97.4%, 96.1% and 98.7%, respectively, and for other CTD baseline patients, 97.9%, 98.7% and 97.1%, respectively. (**e**) Key wavenumbers responsible for GA-LDA model class separation at baseline shown on the original spectra. (**f**) Key wavenumbers responsible for GA-LDA model class separation at follow-up shown on the original spectra. (**g**) ROC analysis for Monte Carlo cross-validation at baseline showed an AUC of 1.00. (**h**) ROC analysis for Monte Carlo cross-validation at follow-up showed an AUC of 0.974.

**Figure 3:**
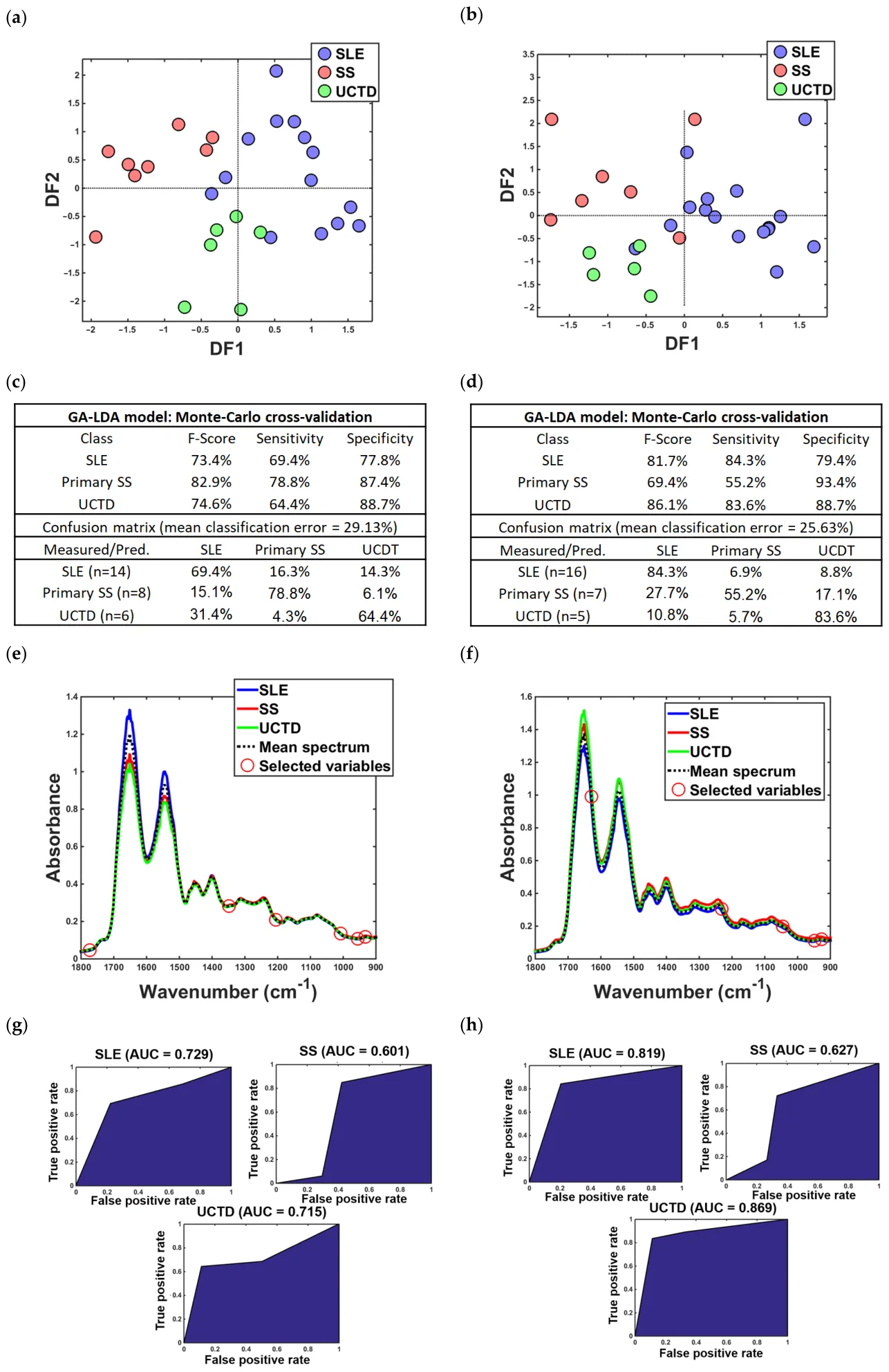
GA-LDA classification analysis showing clustering for SLE, SS and UCTD patients at (**a**) baseline and (**b**) follow-up time points. (**c**) Accuracy, sensitivity and specificity calculated for the Monte Carlo cross-validation data at baseline: 73.4%, 69.4% and 77.8%, respectively, for SLE; 82.9%, 78.8% and 87.4%, respectively, for SS; and 74.6%, 64.4% and 88.7%, respectively, for UCTD. (**d**) Accuracy, sensitivity and specificity calculated for the Monte Carlo cross-validation data at follow-up: 81.7%, 84.3% and 79.4%, respectively, for SLE; 69.4%, 55.2% and 93.4%, respectively, for SS; and 86.1%, 83.6% and 88.7%, respectively, for UCTD. (**e**) Average fingerprint spectra per class at baseline. (**f**) Average fingerprint spectra per class at follow-up. (**g**) ROC for Monte Carlo cross-validation GA-LDA model at baseline showed AUCs of 0.729 for SLE, 0.601 for SS, and 0.715 for UCTD. (**h**) ROC for Monte Carlo cross-validation GA-LDA model at follow-up showed AUCs of 0.818 for SLE, 0.627 for SS, and 0.869 for UCTD.

**Table 1:** Demographics of patient cohort.

	SLE/SLE Overlap (n = 16)	SS (n = 8)	UCTD (n = 6)	Overall (n = 30)	*p*-Value *
Demographics					
Male	1 (6.25)	0 (0)	0 (0)	1 (3.33)	~1.0 **
Female	15 (93.75)	8 (100)	6 (100)	29 (96.67)	
Age	47 (23–70)	47 (31–61)	42 (23–85)	46 (23–85)	<0.05 ***
Ethnicity					
White	12 (75)	7 (87.5)	3 (50)	22 (73.33)	0.284
Black	1 (6.25)	0 (0)	3 (50)	4 (13.33)	0.012
Asian	2 (12.5)	1 (12.5)	0 (0)	3 (10)	0.659
Multiple mixed ethnic backgrounds	1 (6.25)	0 (0)	0 (0)	1 (3.33)	0.636
Manifestations					
Anti-dsDNA	3 (18.75)	1 (12.5)	1 (16.67)	5 (16.67)	0.91
Anti-ro52	8 (50)	8 (100)	3 (50)	19 (63.33)	0.135
Anti-ro60	16 (100)	6 (75)	6 (100)	28 (93.33)	0.17
Anti-La	4 (25)	6 (75)	1 (16.67)	11 (36.67)	0.048
Anti-Smith	1 (6.25)	0 (0)	3 (50)	4 (13.33)	0.045
Anti-RNP	4 (25)	0 (0)	3 (50)	7 (23.33)	0.062
Anti-Chromatin	5 (31.25)	0 (0)	3 (50)	8 (26.67)	0.060
Anti-Centromere	0 (0)	0 (0)	0 (0)	0 (0)	--
Anti-Scl70	1 (6.25)	0 (0)	0 (0)	1 (3.33)	0.66
Anti-RNA Pol III	0 (0)	0 (0)	0 (0)	0 (0)	--
	SLE/SLE Overlap Baseline (n = 14) Follow Up (n = 16)	SS Baseline (n = 8) Follow Up (n = 7)	UCTD Baseline (n = 6) Follow Up (n = 5)	Overall Baseline (n = 28) Follow Up (n = 28)	
Medication					
Immunosuppressant Baseline	8 (57.14)	1 (12.5)	0 (0)	9 (32.14)	0.018
Immunosuppressant Follow-up	9 (56.25)	1 (14.28)	0 (0)	10 (35.71)	0.012
Anti-malarial Baseline	9 (64.28)	5 (62.5)	1 (16.67)	15 (53.57)	0.164
Anti-malarial Follow-up	11 (68.75)	4 (57.14)	1 (20)	16 (57.14)	0.080
Disease Activity Score					
High DAS Baseline	2 (14.28)	3 (37.5)	1 (16.67)	6 (21.42)	0.43
High DAS Follow-up	7 (43.75)	3 (48.85)	1 (20)	11 (39.28)	0.40

* *p*-value calculated based on a χ^2^ test. ** *p*-value estimated based on Fisher’s exact test. *** *p*-value estimated based on a Kruskal–Wallis test. *p* < 0.05 is considered statistically significant.

**Table 2:** Key wavenumbers responsible for classification of HC, all CTD (baseline) and all CTD (follow-up) patient groups, with corresponding biomolecular associations and FTIR assignments [26].

Wavenumber (cm^−1^)	Biomolecule	Tentative Assignment
975	Carbohydrate	C-O stretching (carbohydrate-associated)
1016	Carbohydrate	C-C and C-O stretching (carbohydrate-associated)
1061	Carbohydrate	C-O stretching (carbohydrate-associated)
1140	Carbohydrate	C-O-C stretching (polysaccharide-associated)
1154	Carbohydrate	C-O-C stretching (carbohydrate-associated)
1189	DNA	Phosphate group vibrations (PO_2_^−^ stretching)
1196	DNA	Phosphate group vibrations (PO_2_^−^ stretching)
1268	Protein	Amide III band (C-N stretching, N-H bending)
1323	Protein	CH_2_ wagging and side-chain vibrations of serum proteins
1348	Protein	CH deformation and amide III-related vibrations
1438	Lipid	CH_2_ scissoring (lipid-associated)
1442	Lipid	CH_2_ bending (lipid-associated)
1454	Lipid	CH_2_ deformation (lipid-associated)
1570	Protein	Amide II band (N-H bending and C-N stretching), possible minor contributions from aromatic ring vibrations
1608	Protein	Amide I band (protein backbone vibrations)
1615	Protein	Amide I band (protein-associated)
1631	Protein	Amide I band (protein-associated)
1653	Protein	Amide I band (predominantly serum proteins, e.g., albumin)
1674	Protein	Amide I band (protein-associated)
1679	Protein	Amide I band (protein-associated)
1685	Protein	Amide I band (protein-associated)
1712	Lipid	C=O stretching (ester carbonyl groups in lipids)

**Table 3:** Key wavenumbers responsible for classification of SLE vs. other CTD patient groups at baseline and follow-up, with corresponding biomolecular associations and FTIR assignments [26].

Wavenumber (cm^−1^)	Molecular Group	Tentative Assignment	Time Point
903	DNA	Phosphate backbone vibrations (phosphodiester groups)	Baseline
907	DNA	Phosphate backbone vibrations (phosphodiester groups)	Baseline
912	DNA	Phosphate backbone vibrations (phosphodiester groups)	Baseline
951	DNA	Phosphate-related vibrations (PO_4_^3−^/PO_2_^−^)	Follow-up
984	Carbohydrate	C-O stretching (carbohydrate-associated)	Follow-up
985	Carbohydrate	C-O stretching (carbohydrate-associated)	Follow-up
990	Carbohydrate	C-O and C-C vibrations (carbohydrate-associated)	Follow-up
1081	DNA	Symmetric phosphate stretching vibrations	Follow-up
1125	Carbohydrate	C-O stretching (polysaccharide-associated)	Baseline
1144	Carbohydrate	C-O-C and ring vibrations (carbohydrate-associated)	Baseline
1202	DNA	Asymmetric phosphate stretching vibrations	Follow-up
1233	Protein/DNA	Overlapping amide III and phosphate vibrations	Baseline
1253	Protein	Amide III band (C-N stretching, N-H bending)	Follow-up
1300	Protein	Amide III band and protein side-chain vibrations	Follow-up
1339	Carbohydrate	C-O and ring vibrations (carbohydrate-associated)	Baseline
1349	Lipid	CH_2_ wagging vibrations (lipid contributions)	Baseline
1353	Protein	Protein side-chain and amide III-related vibrations	Follow-up
1442	Lipid	CH_2_ bending (lipid-associated)	Follow-up
1453	Lipid	CH_3_/CH_2_ deformation (lipid-associated)	Baseline
1576	Protein	Amide II band (protein backbone), possible minor aromatic contributions	Follow-up
1662	Protein	Amide I band (protein backbone; predominantly serum proteins, e.g., albumin)	Baseline
1723	Lipid	C=O stretching (ester carbonyl groups in lipids)	Baseline
1766	Lipid	C=O stretching (lipid-associated ester groups)	Follow-up
1775	Lipid	C=O stretching (lipid-associated ester groups)	Baseline

**Table 4:** Key wavenumbers responsible for classification of SLE vs. SS vs. UCTD patient groups at baseline and follow-up, with corresponding biomolecular associations and FTIR assignments [26].

Wavenumber (cm^−1^)	Tentative Assignment	Biomolecular Class	Source
927	Phosphodiester backbone vibrations (nucleic acids)	DNA	Follow-up
933	Phosphodiester backbone vibrations (nucleic acids)	DNA	Baseline
948	Phosphate vibrations (PO_2_^−^/PO_4_^3−^)	DNA	Follow-up
956	Phosphate vibrations (PO_2_^−^/PO_4_^3−^)	DNA	Baseline
1008	Protein/nucleic acid contributions	Protein/DNA	Baseline
1045	Carbohydrates (C-O stretching)	Carbohydrate	Follow-up
1205	Overlapping carbohydrate (C-O, C-O-C) and phosphate vibrations	Carbohydrate/DNA	Baseline
1231	PO_2_^−^ asymmetric vibration	DNA	Follow-up
1348	CH_2_ wagging (lipid/protein contributions)	Lipid/Protein	Baseline
1629	Amide I band (protein backbone vibrations)	Protein	Follow-up
1774	C=O stretching (ester carbonyl groups in lipids)	Lipid	Baseline

## Data Availability

Raw data along with appropriate class identifiers will be uploaded to the publicly accessible data repository Figshare (https://doi.org/10.6084/m9.figshare.6025748).

## Supplementary Materials

The following supporting information can be downloaded at https://www.mdpi.com/article/10.3390/biomedicines14102191/s1. Figure S1: Two outliers’ spectra identified in the dataset. Table S1: Monte Carlo cross-validation accuracy (20% of samples out, 1000 iterations) for different classification algorithms applied to classify between healthy controls (HC), SLE baselines and SLE follow-ups. PCA-LDA: Principal component analysis with linear discriminant analysis; PLS-DA: partial least squares discriminant analysis; iPLS-DA: interval partial least squares discriminant analysis; SPA-LDA: successive projections algorithm with linear discriminant analysis; GA-LDA: genetic algorithm with linear discriminant analysis. Table S2: Statistical analysis was carried out using a Kruskal–Wallis test, where *p*-values below 0.05 were considered statistically significant. Data comparisons were for sex, age, ethnicity, disease activity score (DAS), treatments (anti-malarials and biologic immunosuppressants), and the following antibodies: anti-double stranded DNA (dsDNA), anti-Ro52, anti-Ro60, anti-La, anti-Sm, anti-RNP, anti-Chromatin, anti-Centromere, anti-Scl70, and anti-Jo-1. Table S3: Comparison of wavenumber average absorbance intensities for key discriminatory wavenumbers between HC, all CTD baseline and all CTD follow-up patients. The absorbances for all selected wavenumbers by the GA-LDA model are higher for the SLE follow-up group and lower for the HC group. Table S4: Comparison of wavenumber average absorbance intensities for key discriminatory wavenumbers between SLE/SLE overlap patients and other CTDs at baseline. The absorbance for all selected wavenumbers by the GA-LDA model is higher for the other CTDs group. Table S5: Comparison of wavenumber average absorbance intensities for key discriminatory wavenumbers between SLE/SLE overlap patients and other CTDs at follow-up. The absorbance for all selected wavenumbers by the GA-LDA model is higher for the other CTDs group. Table S6: Comparison of wavenumber average absorbance intensities for key discriminatory wavenumbers between SLE/SLE overlap, SS and UCTD patients at baseline. The absorbance for all selected wavenumbers by the GA-LDA model is higher for the primary SS group. Table S7: Comparison of wavenumber average absorbance intensities for key discriminatory wavenumbers between SLE/SLE overlap, SS and UCTD patients at follow-up. The absorbance for all selected wavenumbers by the GA-LDA model is higher for the primary SS group, with the exception of 1629 cm^−1^.

## Abbreviations

The following abbreviations are used in this manuscript:

ANA	Antinuclear Antibodies
ARC NWC	Applied Research Collaboration North West Coast
AUC	Area Under Curve
BRC	Biomedical Research Centre
cfDNA	Cell-free DNA
CTD	Connective Tissue Disease
DAS	Disease Activity Score
DNA	Deoxyribonucleic Acid
dsDNA	Double-stranded DNA
FTIR	Fourier-Transform Infrared
GA	Genetic Algorithm
GA-LDA	Genetic Algorithm–Linear Discriminant Analysis
HC	Healthy Control
HCRW	Health and Care Research Wales
HDL	High-density Lipoprotein
HEp-2	Human Epithelial Cell Line
HRA	Health Research Authority
iPLS-DA	Interval Partial Least Squares Discriminant Analysis
LDA	Linear Discriminant Analysis
LDL	Low-density Lipoprotein
LEAP	Lupus Extended Autoimmune Phenotype
MCTD	Mixed Connective Tissue Disease
MDSS	Medical Decision Support Software
NIHR	National Institute for Health and Care Research
PCA-LDA	Principal Component Analysis with Linear Discriminant Analysis
PLS-DA	Partial Least Squares Discriminant Analysis
PTM	Post-Translational Modification
qPCR	Quantitative PCR
ROC	Receiver Operating Characteristics
SARD	Systemic Autoimmune Rheumatic Diseases
SLE	Systemic lupus erythematosus
SPA-LDA	Successive Projections Algorithm with Linear Discriminant Analysis
SS	Sjögren’s Syndrome
UCTD	Undifferentiated Connective Tissue Disease
VLDL	Very Low-density Lipoprotein
