# Supplementary material for: Towards a Novel Diagnostic Tool for Autoimmune Rheumatic Diseases: Classification of SLE Using FTIR Spectroscopy and Machine Learning
Source: Biomedicines. 2026 Sep 28;14(10):2191. doi: 10.3390/biomedicines14102191 (PMC13701543; doi:10.3390/biomedicines14102191)
Supplement: Supplementary file 1 [file biomedicines-14-02191-s001.zip › biomedicines-4547427-supplementary.pdf]

Supplementary Information

Methods

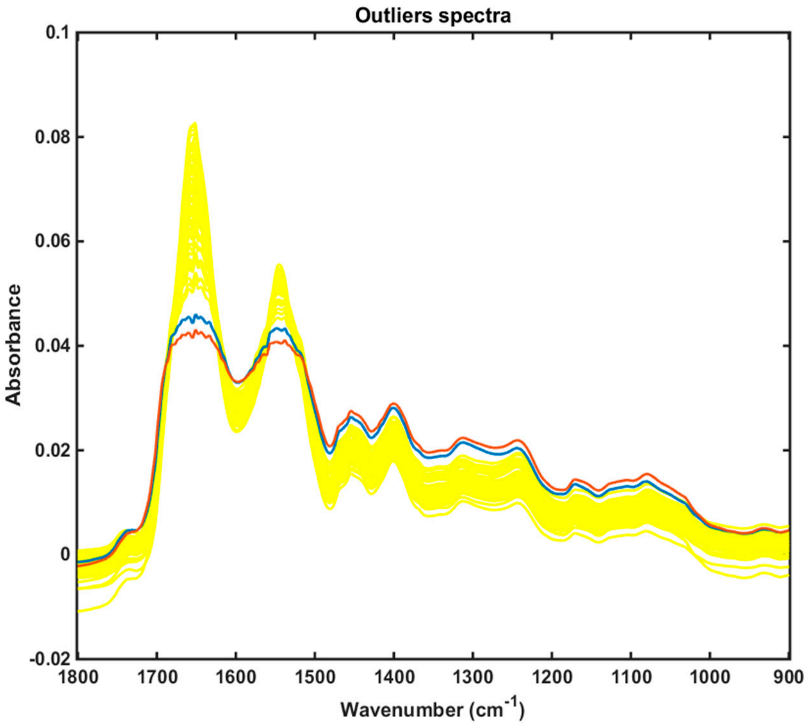

Figure S1. Two outliers' spectra identified in the dataset.

Table S1. Monte Carlo cross-validation accuracy (20% of samples out, 1000 iterations) for different classification algorithms applied to classify between healthy controls (HC), SLE baselines and SLE follow ups. PCA-LDA: principal component analysis with linear discriminant analysis, PLS-DA: partial least squares discriminant analysis, iPLS-DA: interval partial least squares discriminant analysis; SPA-LDA: successive projections algorithm with linear discriminant analysis; GA-LDA: genetic algorithm with linear discriminant analysis.

**Model**	**Parameters***	**% HC**	**% SLE baseline**	**% SLE follow up**	**% Average**
PCA-LDA	16 PCs (99.67%)	28.0%	84.5%	80.9%	64.5%
PLS-DA	16 LVs (99.56%)	53.8%	86.8%	88.8%	76.4%
iPLS-DA	13 LVs (98.74%)	77.3%	88.5%	83.6%	83.1%
SPA-LDA	16 variables	23.6%	84.8%	73.7%	60.7%
GA-LDA	22 variables	92.1%	99.1%	97.1%	96.1%

*Explained variance within parentheses. PCs: principal components. LVs: latent variables.

Results

Table S2. Statistical analysis was carried out using a Kruskal–Wallis test, where p-values below 0.05 were considered statistically significant. Data comparisons were for sex, age, ethnicity, disease activity score, treatments (anti-malarials and biologic immunosuppressants), and the following antibodies: anti-double stranded DNA (dsDNA), anti-Ro52, anti-Ro60, anti-La, anti-Sm, anti-RNP, anti-Chromatin, anti-Centromere, anti-Scl70, and anti-Jo-1.

Parameter	Groups	p-value
Sex	SLE = 30 F (93.7%), 2 M (6.3%)<br>Sjögren's syndrome = 16 F (100%)<br>UCTD = 12 F (100%)<br><br>Total<br>Females (F) = 58 (96.7%)<br>Males (M) = 2 (3.3%)	0.4106
Serology (autoantibodies)		
anti_dsdna	SLE = 7 P (21.9%), 25 N (78.1%)<br>Sjögren's syndrome = 2 P (12.5%), 14 N (87.5%)<br>UCTD = 2 P (16.7%), 10 N (83.3%)<br><br>Total<br>Positives (P) = 11 (18.3%)<br>Negative (N) = 49 (81.7%)	0.7250
anti_ro52	SLE = 9 P (52.9%), 8 N (47.1%)<br>Sjögren's syndrome = 8 P (100%), 0 N (0.0%)<br>UCTD = 3 P (50.0%), 3 N (50.0%)<br><br>Total<br>Positives (P) = 20 (64.5%)<br>Negatives (N) = 11 (35.5%)	0.0563
anti_ro60	SLE = 17 P (100%), 0 N (0.0%)<br>Sjögren's syndrome = 6 P (75.0%), 2 N (25.0%)<br>UCTD = 6 P (100%), 0 N (0.0%)<br><br>Total<br>Positives (P) = 29 (93.5%)<br>Negatives (N) = 2 (6.5%)	0.0511
anti_la	SLE = 4 P (23.5%), 13 N (76.5%)<br>Sjögren's syndrome = 5 P (62.5%), 3 N (37.5%)<br>UCTD = 1 P (16.7%), 5 N (83.3%)<br><br>Total<br>Positives (P) = 10 (32.3%)<br>Negatives (N) = 21 (67.7%)	0.1075
anti_sm	SLE = 1 P (6.3%), 15 N (93.7%)<br>Sjögren's syndrome = 0 P (0.0%), 8 N (100%)<br>UCTD = 3 P (50.0%), 3 N (50.0%)<br><br>Total<br>Positives (P) = 4 (13.3%)<br>Negatives (N) = 26 (86.7%)	0.0135*
anti_rnp	SLE = 4 P (23.5%), 13 N (76.5%)<br>Sjögren's syndrome = 0 P (0.0%), 8 N (100%)	0.0924

	UCTD = 3 P (50.0%), 3 N (50.0%)<br><br>Total<br>Positives (P) = 7 (22.6%)<br>Negatives (N) = 24 (77.4%)	
anti_chromatin	SLE = 5 P (29.4%), 12 N (70.6%)<br>Sjögren's syndrome = 0 P (0.0%), 8 N (100%)<br>UCTD = 3 P (50.0%), 3 N (50.0%)<br><br>Total<br>Positives (P) = 8 (25.8%)<br>Negatives (N) = 23 (74.2%)	0.1013
anti_centromere	SLE = 0 P (0.0%), 17 N (100%)<br>Sjögren's syndrome = 0 P (0.0%), 8 N (100%)<br>UCTD = 0 P (0.0%), 6 N (100%)<br><br>Total<br>Positives (P) = 0 (0%)<br>Negatives (N) = 31 (100%)	--
anti_scl70	SLE = 1 P (5.9%), 16 N (94.1%)<br>Sjögren's syndrome = 0 P (0.0%), 8 N (100%)<br>UCTD = 0 P (0.0%), 6 N (100%)<br><br>Total<br>Positives (P) = 1 (3.2%)<br>Negatives (N) = 30 (96.8%)	0.6625
Ethnicity		
ethnic_cat	SLE = 12 W (75.0%), 1 B (6.25%), 2 A (12.5%), 1 M (6.25%)<br>Sjögren's syndrome = 7 W (87.5%), 1 A (12.5%)<br>UCTD = 3 W (50.0%), 3 B (50.0%)<br><br>Total<br>White (W) = 22 (73.3%)<br>Black (B) = 4 (13.3%)<br>Asian (A) = 3 (10.0%)<br>Mixed ethnicity (M) = 1 (3.3%)	0.4854
Disease Activity Score		
high_das	SLE = 9 Y (28.1%), 23 N (71.9%)<br>Sjögren's syndrome = 6 Y (37.5%), 10 N (62.5%)<br>UCTD = 2 Y (16.7%), 10 N (83.3%)<br><br>Total<br>Yes (Y) = 17 (28.3%)<br>No (N) = 43 (71.7%)	0.4861
Treatment		
anti_malaria	SLE = 22 Y (68.7%), 10 N (31.3%)<br>Sjögren's syndrome = 10 Y (62.5%), 6 N (37.5%)<br>UCTD = 2 Y (16.7%), 10 N (83.3%)<br><br>Total<br>Yes (Y) = 34 (56.7%)<br>No (N) = 26 (43.3%)	0.0075*
immunosuppressant	SLE = 18 Y (56.3%), 14 N (43.7%)<br>Sjögren's syndrome = 2 Y (12.5%), 14 N (87.5%)<br>UCTD = 0 Y (0.0%), 12 N (100%)<br><br>Total<br>Yes (Y) = 20 (33.3%)<br>No (N) = 40 (66.7%)	2.73e-04*
Age		
age_rounded	SLE = 46.9 ± 12.3<br>Sjögren's syndrome = 47.0 ± 11.2	0.1248

	UCTD = 41.7 ± 21.6	

Table S3. Comparison of wavenumber average absorbance intensities for key discriminatory wavenumbers between HC, all CTD baseline and all CTD follow up patients. The absorbance for all selected wavenumbers by the GA-LDA model are higher for the SLE follow up group and lower for the HC group.

**Average absorbance**					
Selected variables (cm^{-1})	HC	All CTD baseline	All CTD follow up	Lower	Higher
975	0.0989	0.1129	0.1155	HC	SLE follow up
1016	0.1273	0.1470	0.1492	HC	SLE follow up
1061	0.1802	0.2115	0.2142	HC	SLE follow up
1140	0.1669	0.1929	0.1958	HC	SLE follow up
1154	0.1794	0.2072	0.2105	HC	SLE follow up
1189	0.1715	0.1953	0.1990	HC	SLE follow up
1196	0.1731	0.1977	0.2016	HC	SLE follow up
1268	0.2581	0.2956	0.3046	HC	SLE follow up
1323	0.2645	0.3035	0.3116	HC	SLE follow up
1348	0.2439	0.2821	0.2882	HC	SLE follow up
1438	0.3246	0.3741	0.3886	HC	SLE follow up
1442	0.3379	0.3898	0.4048	HC	SLE follow up
1454	0.3543	0.4084	0.4237	HC	SLE follow up
1570	0.5831	0.6622	0.7166	HC	SLE follow up
1608	0.4892	0.5645	0.5967	HC	SLE follow up
1615	0.5588	0.6457	0.6883	HC	SLE follow up
1631	0.8249	0.9319	1.0561	HC	SLE follow up
1653	1.0390	1.1259	1.3450	HC	SLE follow up
1674	0.8071	0.9058	1.0348	HC	SLE follow up
1679	0.7149	0.8115	0.9106	HC	SLE follow up
1685	0.5829	0.6707	0.7353	HC	SLE follow up
1712	0.1282	0.1487	0.1493	HC	SLE follow up

Table S4. Comparison of wavenumber average absorbance intensities for key discriminatory wavenumbers between SLE/SLE overlap patients and other CTDs at baseline. The absorbance for all selected wavenumbers by the GA-LDA model are higher for the Other CDTs group.

**Average absorbance**				
Selected variables (cm^{-1})	SLEs	Other CTDs	Lower	Higher
903	0.1031	0.1074	SLEs	Other CTDs
907	0.1017	0.1058	SLEs	Other CTDs
912	0.1012	0.1052	SLEs	Other CTDs

1125	0.1915	0.1973	SLEs	Other CTDs
1144	0.1772	0.1831	SLEs	Other CTDs
1233	0.2749	0.2880	SLEs	Other CTDs
1339	0.2595	0.2730	SLEs	Other CTDs
1349	0.2564	0.2696	SLEs	Other CTDs
1453	0.3707	0.3930	SLEs	Other CTDs
1662	0.9977	1.1290	SLEs	Other CTDs
1723	0.0990	0.1014	SLEs	Other CTDs
1775	0.0453	0.0453	SLEs	Other CTDs*

*Difference in the 5th decimal place

Table S5. Comparison of wavenumber average absorbance intensities for key discriminatory wavenumbers between SLE/SLE overlap patients and other CTDs at follow up. The absorbance for all selected wavenumbers by the GA-LDA model are higher for the Other CDTs group.

**Average absorbance**				
Selected variables (cm^{-1})	SLEs	Other CTDs	Lower	Higher
951	0,0957	0,1090	SLEs	Other CTDs
984	0,1028	0,1164	SLEs	Other CTDs
985	0,1030	0,1166	SLEs	Other CTDs
990	0,1050	0,1187	SLEs	Other CTDs
1081	0,2052	0,2303	SLEs	Other CTDs
1202	0,1801	0,2043	SLEs	Other CTDs
1253	0,2729	0,3114	SLEs	Other CTDs
1300	0,2761	0,3136	SLEs	Other CTDs
1353	0,2459	0,2817	SLEs	Other CTDs
1442	0,3434	0,3929	SLEs	Other CTDs
1576	0,5535	0,6349	SLEs	Other CTDs
1766	0,0470	0,0544	SLEs	Other CTDs

Table S6. Comparison of wavenumber average absorbance intensities for key discriminatory wavenumbers between SLE/SLE overlap, SS and UCTD patients at baseline. The absorbance for all selected wavenumbers by the GA-LDA model are higher for the Primary SS group.

**Average absorbance**

Selected variables (cm^{-1})	SLE	Primary SS	UCTD	Lower	Higher
939	0.1154	0.1236	0.1168	SLE	Primary SS
979	0.1052	0.1124	0.1072	SLE	Primary SS
1093	0.1321	0.1379	0.1350	SLE	Primary SS
1151	0.2070	0.2159	0.2088	SLE	Primary SS
1683	0.2807	0.2875	0.2798	UCTD	Primary SS
1762	0.0443	0.0507	0.0485	SLE	Primary SS

Table S7. Comparison of wavenumber average absorbance intensities for key discriminatory wavenumbers between SLE/SLE overlap, SS and UCTD patients at follow up. The absorbance for all selected wavenumbers by the GA-LDA model are higher for the Primary SS group, with the exception of 1629 cm-1.

**Average absorbance**					
Selected variables (cm^{-1})	SLE	Primary SS	UCTD	Lower	Higher
927	0.1135	0.1349	0.1199	SLE	Primary SS
948	0.1058	0.1258	0.1124	SLE	Primary SS
1045	0.1893	0.2162	0.1992	SLE	Primary SS
1231	0.2891	0.3367	0.3140	SLE	Primary SS
1629	0.9418	1.0503	1.0596	SLE	UCTD
